# Lentiform Nucleus Hyperechogenicity in Parkinsonian Syndromes: A Systematic Review and Meta-Analysis with Consideration of Molecular Pathology

**DOI:** 10.3390/cells9010002

**Published:** 2019-12-18

**Authors:** Daniel Richter, Aristeidis H. Katsanos, Christoph Schroeder, Georgios Tsivgoulis, George P. Paraskevas, Thomas Müller, Andrei V. Alexandrov, Ralf Gold, Lars Tönges, Christos Krogias

**Affiliations:** 1 Department of Neurology, St. Josef-Hospital, Ruhr-University Bochum, 44791 Bochum, Germany; daniel.richter-c34@rub.de (D.R.); ar.katsanos@gmail.com (A.H.K.); christoph.schroeder@rub.de (C.S.); ralf.gold@rub.de (R.G.); lars.toenges@rub.de (L.T.); 2 2nd Department of Neurology, National and Kapodistrian University of Athens, 15344 Athens, Greece; tsivgoulisgiorg@yahoo.gr; 3 Department of Neurology, The University of Tennessee Health Science Center, Memphis, TN 38163, USA; avalexandrov@att.net; 4 1st Department of Neurology, Cognitive and Movement Disorders Clinic and Unit of Neurochemistry and Biological Markers, School of Medicine, National and Kapodistrian University of Athens, Eginition Hospital, 11528 Athens, Greece; gparask@med.uoa.gr; 5 Department of Neurology, Alexianer St. Joseph Berlin-Weißensee, 13088 Berlin, Germany; Th.Mueller@alexianer.de; 6 Neurodegeneration Research, Protein Research Unit Ruhr (PURE), Ruhr University Bochum, 44791 Bochum, Germany

**Keywords:** transcranial sonography, Parkinson’s disease, nucleus lentiformis, hyperechogenicity

## Abstract

The hyperechogenicity of the substania nigra (SN) has been established as a valid finding in patients with Parkinson’s disease (PD), probably caused by an increased tissue iron concentration in the SN. The application of transcranial sonography (TCS) has been investigated for further echogenic basal ganglia alterations in patients with extrapyramidal movement disorders. Compared to PD, a hyperechogenic nucleus lentiformis (LN) has been reported to appear more frequently in atypical parkinsonian syndromes (aPS) such as the parkinsonian phenotype of multiple system atrophy (MSA-P) or the progressive supranuclear palsy (PSP). As the evidence providing study sizes are small, we conduct the first meta-analysis of the prevalence of LN hyperechogenicity in PD and aPS. We search for available studies providing prevalence of LN hyperechogenicity in patients with PD and aPS (MSA-P and PSP) detected by TCS in MEDLINE and SCOPUS databases. We calculate the prevalence rates of LN hyperechogenicity detection in patients with clinical diagnosis of PD vs. aPS under the random-effects model. We include a total of 1330 patients, 1091 PD and 239 aPS (MSA-P and PSP). We find a significantly higher prevalence of LN hyperechogenicity in aPS (76%, 95% CI: 0.62-0.88) compared to PD (16%, 95% CI: 0.10-0.23). After proving a higher prevalence of LN hyperechogenicity in aPS compared to PD, its histopathological cause needs to be investigated. Furthermore, its full diagnostic accuracy and the qualification to serve as a risk factor for MSA-P and PSP should also be questioned in future studies.

## 1. Introduction

Transcranial sonography (TCS) in Parkinson’s disease (PD) has been increasingly applied over the last two decades and has proven to be a very helpful tool in the diagnostic process and risk stratification of extrapyramidal movement disorders [1]. Substantia nigra (SN) hyperechogenicity could be identified as a typical finding in patients with idiopathic Parkinson’s disease (PD), allowing a clear distinction between PD and healthy controls [2], while a distinction between idiopathic and atypical parkinsonian syndromes (aPS) is more difficult [3]. The meta-analysis by Shafieesabet et al. [4] found a prevalence of SN hyperechogenicity in 84% of PD patients and only in 28% of aPS patients. Reasons for hyperechogenic alterations in the SN are assumed to be caused by an increase in the amount of tissue iron content [5]. Apart from the SN, several other structures in the brain have been examined by TCS in extrapyramidal movement disorders [5,6]. A hyperechogenicity of the nucleus lentiformis (LN) was found to appear more frequently in patients with aPS, especially in patients with the parkinsonian phenotype of multiple system atrophy (MSA-P), or in patients with progressive supranuclear palsy (PSP) [7]. Thus, LN hyperechogenicity has been considered as a promising marker of aPS, although the scientific evidence of this observation was based on several studies with only a small number of patients. Therefore, we perform the first meta-analysis to date on LN hyperechogenicity prevalence in PD and aPS.

## 2. Materials and Methods

The present systematic review and meta-analysis was conducted according to the Preferred Reporting Items of Systematic Reviews and Meta-Analyses (PRISMA) statement [8]. We searched for available studies providing prevalence of LN hyperechogenicity in patients with PD and aPS detected by TCS, which is defined as any echogenic signal at the anatomical site of the LN in comparison to the surrounding white matter that can be visually assessed [5]. Disease diagnosis was based on the respective clinical diagnostic criteria for PD (UK Brain Bank Criteria) [9] and PSP [10]. MSA-P patients met the international criteria for MSA [11] and in one study [12] for clinically probable MSA [13]. The literature search in the MEDLINE and SCOPUS databases was performed by two independent reviewers (D.R. and A.H.K.) using the following terms in combination: “nucleus lentiformis”, “basal ganglia”, “transcranial sonography”, “transcranial ultrasound”, “Parkinson”, “multiple system atrophy”, “progressive supranuclear palsy”, “movement disorder”. The complete algorithm used in the MEDLINE database search is available in the online supplement (File S1). No language or other search restriction was applied. The last literature search was performed on April 4th, 2019.

We calculated the rates of LN hyperechogenicity detection by TCS by dividing the number of cases (patients with LN hyperechogenicity) by the total number of patients receiving TCS. After the overall analysis, we performed subgroup analyses according to the clinical diagnosis of PD vs. aPS. For all proportion analyses we implemented the variance-stabilizing double arcsine transformation [14]. The random-effects model (DerSimonian and Laird) was used to calculate the pooled estimates in both the overall and subgroup analyses. We assessed heterogeneity between studies with the Cochrane Q and I^2^ statistics. For the subgroup analyses we used a standard test for heterogeneity across subgroup results to investigate potential differences between subgroups. All statistical analyses were performed with the use of the Stata Statistical Software Release 13 for Windows (College Station, TX, StataCorp LP).

## 3. Results

**Figure 1:**
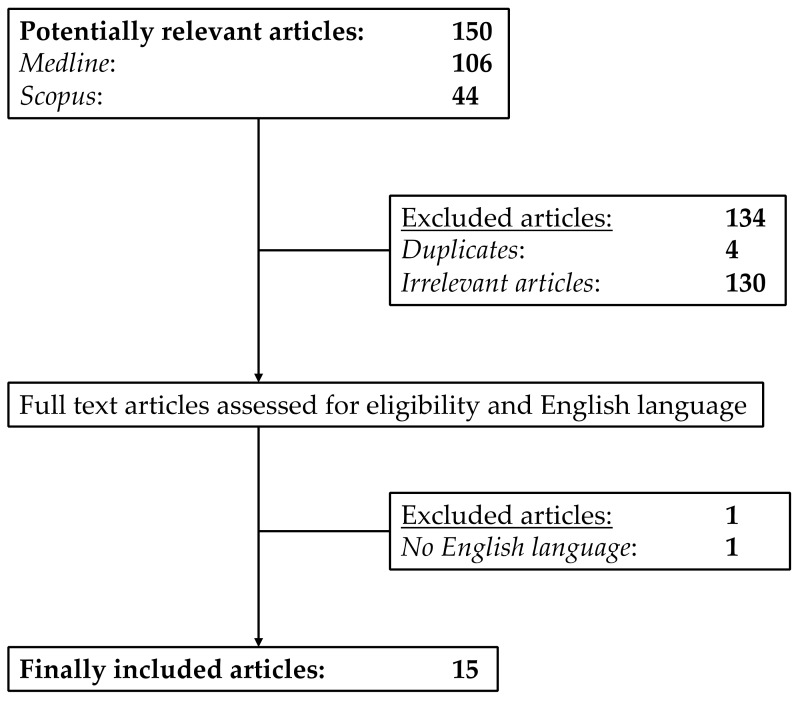
Flow chart of the selection process for the included studies.

From research on MEDLINE and SCOPUS we found 150 potentially relevant articles. Figure 1 shows the selection process visualized as a flow chart. After assessing the abstracts, 135 articles were excluded.

The remaining 15 [7,12,15,16,17,18,19,20,21,22,23,24,25,26,27] articles were included in our meta-analysis. These 15 articles provided the data of overall 1330 patients with a sufficient bone window subdivided into 1091 PD and 239 aPS (MSA-P and PSP) patients. The main characteristics of the included studies are summarized in Table 1.

**Figure 2:**
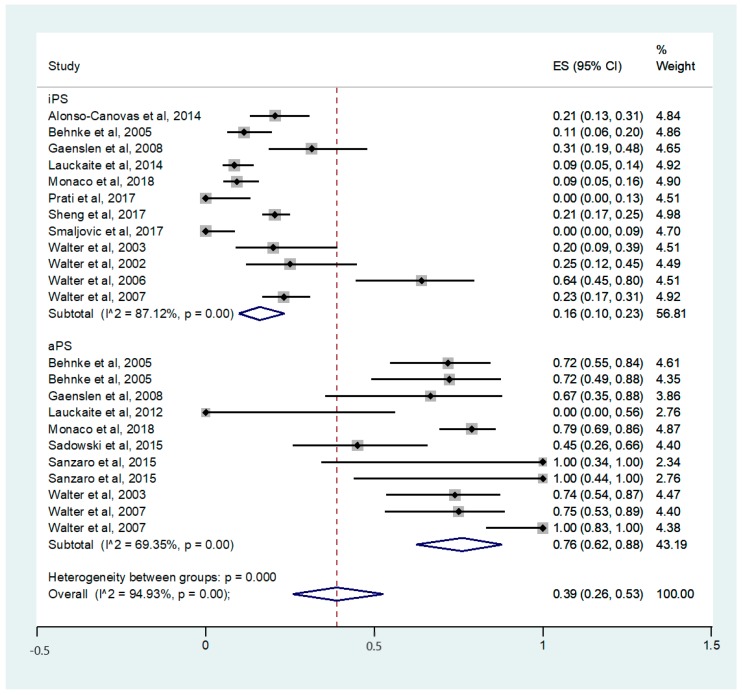
Forest plots of the studies included for the calculation of prevalence of LN hyperechogenicity in PD and aPS. PD = Parkinson’s disease; aPS = atypical parkinsonian syndromes.

For the PD group, the prevalence of LN hyperechogenicity was calculated to 16% (95% CI: 0.10-0.23) and ranged from 0% to 64%. In the aPS group, the corresponding prevalence was 76% (95% CI: 0.62–0.88) with a range from 0% to 100%. Heterogenicity was substantial for both groups (PD: I^2^ =8 7.08%, aPS: I^2^ = 69.35%). Figure 2 demonstrates the forest plots of the studies included.

## 4. Discussion

The results of this meta-analysis demonstrate the significant difference regarding the prevalence of LN hyperechogenicity in patients with aPS compared to patients with PD. This strongly indicates that this finding is a valid and helpful marker in the discrimination of parkinsonian syndromes. A hyperechogenic lentiform nucleus frequently occurs in MSA-P and PSP, while it is an uncommon feature in PD. Additionally, the prevalence of LN hyperechogenicity in the healthy population is assumed to be low (7.9% for marked LN hyperechogenicity), but studies specifically targeting this question are lacking [19,28]. Until today, the distinction of PD from MSA-P and PSP is not always easy, especially in early disease. The diagnostic criteria for these different disorders are still based on clinical findings [11,29,30] and, despite the presence of some useful imaging markers [31], biomarkers with high diagnostic accuracy, especially in the early stages, are generally lacking. Therefore, the sonographic evaluation of the LN could be a very helpful tool to identify patients with aPS in everyday practice. Moreover, the combination of the sonographic evaluation of the LN and SN could be a promising tool to distinguish between PD (SN hyperechogenicity without LN hyperechogenicity) and aPS (LN hyperechogenicity without SN hyperechogenicity).

TCS is a rapid, low-cost, non-invasive and safe examination with a very good and reliable interrater agreement. The SN hyperechogenicity has been identified as a risk factor to develop PD [1,32]. Despite all efforts, the correct diagnosis of MSA-P or PSP presenting as sole parkinsonism in the early stages may be difficult and may require an extensive diagnostic approach [33]. The detection of early stages is of major interest because new, potentially disease-modifying therapies are entering clinical trials. Whether LN hyperechogenicity could further serve as risk factor or prodromal marker for MSA-P or PSP remains to be evaluated in future studies.

Based on animal and post-mortem studies, it has been suggested that SN hyperechogenicity in TCS is caused by an increase of the iron concentration in the SN [34,35,36,37]. An elevated cellular iron content was shown to be a potential damaging factor for nigral neurons [38,39]. Until now, it is unclear whether the increased tissue iron accumulation in the SN is the primary cause or a secondary consequence of the neuronal cell degeneration. On the one hand, there is evidence that iron homeostasis could have a causal link to neurodegeneration in different diseases [40]. In a postmortem study there are further hints underlining this finding. In three subjects, Zecca et al. [41] found an increase in the tissue iron concentration in a preclinical form of PD (incidental Lewy body disease) [42] compared to healthy controls without Lewy bodies and with low SN echogenicity. On the other hand, one of the main cellular functions of neuromelanin is to store iron in the SN. A reduction in neuromelanin levels has been linked to an increase of the SN echogenicity and low neuromelanin levels have been related to a low number of dopaminergic cells [43,44]. Additionally, neuromelanin has been discussed to have neuroprotective effects, especially because of its ability to chelate iron [45]. Thus, the increase of the tissue iron concentration could also be a secondary phenomenon of dopaminergic cell loss. Whether similar mechanisms are involved in LN hyperechogenicity is unknown, but MRI studies indicate different patterns of brain iron accumulation in aPS and PD [46]. Presumably, the increase of iron in specific regions of the brain is caused by the underlying disease pathologies. For PD, an iron overload with its inherent toxicity for dopaminergic neurons of the SN is assumed to play an important role in the disease pathogenesis [40]. Concerning MSA and PSP, pathological studies have demonstrated an increase of iron levels in the putamen and globus pallidus but also in the SN [47,48]. So far, there are no studies investigating the cellular and extracellular changes in PD patients with LN hyperechogenicity. However, an increase in the tissue iron level could possibly cause the hyperechogenic alterations of the LN visualized in TCS. Apart from that, Walter et al. conducted a tissue metal analysis in autopsy brains of 11 patients with Wilson’s disease (WD) in which the LN hyperechogenicity is a common ultrasound finding [49]. Diagnosis of WD was confirmed for all of these WD cases after autopsy and they all showed a LN hyperechogenicity in TCS. The authors found a clear correlation between the LN hyperechogenicity and the putaminal concentration of copper, but not of iron. Future studies should examine the histopathological alterations underlying hyperechogenic LN and its possible association to an increased tissue iron or copper concentration in patients with aPS.

## 5. Limitations

**Figure 3:**
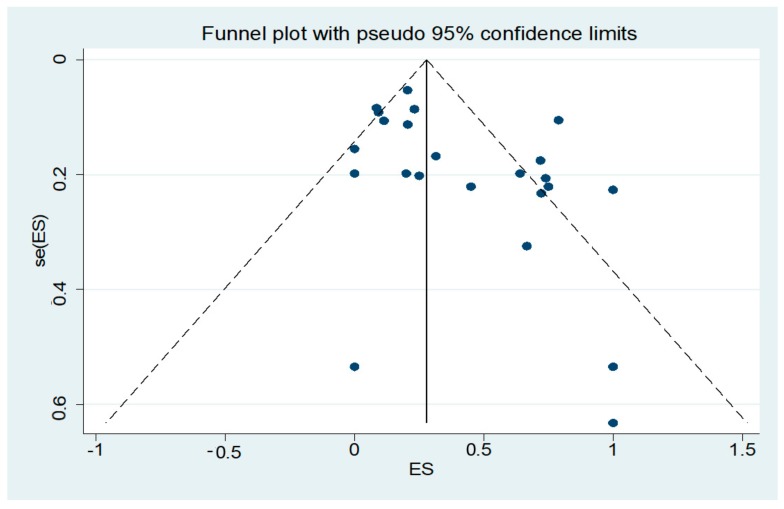
Funnel plot of the studies included in the meta-analysis.

To our knowledge, this is the first meta-analysis assessing the prevalence of LN hyperechogenicity in TCS for patients with PD and aPS (MSA-P or PSP). The literature search and data extraction were thoroughly conducted to avoid any bias, especially to exclude calculation with the same cohorts in case of identical authorships in different studies. Despite the greatest care, this is a general problem for meta-analysis and cannot be completely solved. Figure 3 demonstrates the funnel plot of this meta-analysis. Here, an asymmetric distribution is visible (Egger’s test, p = 0.024). A selection bias by failure to acquire unpublished or non-English data could have had an impact on the appearance of the plot. Additionally, publication bias as well as small study effects are common reasons for an asymmetric distribution of a funnel plot. We further observed a high grade of heterogeneity by meta-regression analyses which can be caused by random variation between the individual studies. We were not able to calculate the diagnostic accuracy because the corresponding values were not available. Furthermore, some studies did not provide the individual numbers of the MSA-P and PSP cases of their aPS cohort or other important information for a specific subgroup analysis were missing. Thus, we could only calculate the common prevalence of LN hyperechogenicity for MSA-P and PSP together. Due to the concept of the underlying studies, we were also not able to adjust potential confounders which are common limitations in observational studies. Additionally, it has to be mentioned that the methodological information of the TCS examination was inconsistently reported. In particular, the number of the TCS investigators and the data of investigator blinding to diagnosis were not always available. Regarding the sonographic equipment, there were slight differences in the applied TCS probe, which is summarized in Table 1.

## 6. Conclusions

This meta-analysis revealed a high prevalence of LN hyperechogenicity in aPS compared to PD, underlining the importance of TCS examination for the diagnostic process of parkinsonian syndromes. From the neuropathological point of view, MSA-P and PSP are distinct diseases [11,29] which makes this finding even more interesting. Therefore, the cellular and extracellular changes that are related to LN hyperechogenicity, its single or combined diagnostic accuracy, and, respective to the evidence of the SN hyperechogenicty, the ability to serve as a risk factor or early biomarker of aPS should be further investigated in future studies.

## Figures and Tables

**Table 1:** Main characteristics of the studies included.

Authors	Year	Country	Center	TCS Device (MHz)	Ultrasound System	PD Cases	aPS Cases (MSA-P/ PSP)	Mean Age (PD/ aPS)
Monaco et al.	2018	Italy	Mono	2–3.5	Sonos 750, Philipps	119	90 (-/-)	66/62
Prati et al.	2017	Italy	Multi	2.5	APLIO 400 Platinum, Toshiba	25	-	-
Sheng et al.	2017	China	Mono	2.5	Sequoia 512, Siemens	356	-	64/-
Smaljovic et al.	2017	Bosnia and Herzegovina	Mono	2.5	EnVisor C HD, Philips	41	-	65/-
Sadowski et al.	2015	Poland	Mono	1–4	Esaote, MyLab 70XVision	-	20 (0/20)	-/60
Sanzaro et al.	2015	Italy	Mono	2.5	General Electric, Logiq 7 Pro	-	5 (2/3)	-/-
Alonso-C. et al.	2014	Spain	Mono	2.5	Xario, Toshiba	78	-	73/-
Laučkaitė et al.	2014	Lithuania	Mono	2-5	Voluson 730, General Electrics Healthcare	141	-	64/-
Laučkaitė et al.	2012	Lithuania	Mono	1, 3–4	Voluson 730, General Electrics Healthcare	-	3 (-/-)	67
Gaenslen et al.	2008	Germany	Mono	2.5	Elegra, Siemens	35	9 (-/-)	-/-
Walter et al.	2007	Germany	Mono	2.5	Elegra, Siemens	134	39 (20/19)	67/68
Walter et al.	2006	Germany	Mono	2.5	Elegra, Siemens	25	-	71/-
Behnke et al.	2005	Germany	Multi	2.5	Elegra, Siemens	88	50 (32/18)	67/66
Walter et al.	2003	Germany	Mono	2.5	Elegra, Siemens	25	23 (-/-)	68/69
Walter et al.	2002	Germany	Mono	2.5	Elegra, Siemens	24	-	69/-

## Supplementary Materials

The following are available online at https://www.mdpi.com/2073-4409/9/1/2/s1. File S1: Complete search algorithm.
