# Supplementary material for: Lentiform Nucleus Hyperechogenicity in Parkinsonian Syndromes: A Systematic Review and Meta-Analysis with Consideration of Molecular Pathology
Source: Cells. 2019 Dec 18;9(1):2. doi: 10.3390/cells9010002 (PMC7016776; doi:10.3390/cells9010002)
Supplement: Supplementary file 1 [file cells-09-00002-s001.pdf]

Date of Search: 3/4/2019

Medline (106 results):

(nucleus lentiformis OR putamen OR globus pallidus OR basal ganglia) AND (transcranial sonography or b-mode ultrasound or transcranial ultrasound) and (parkinson or multiple system atrophy or lewy body dementia or progressive supranuclear palsy or parkinsonian syndrome or movement disorder)

Medline complete algorithm:

(("corpus striatum"[MeSH Terms] OR ("corpus"[All Fields] AND "striatum"[All Fields]) OR "corpus striatum"[All Fields] OR ("nucleus"[All Fields] AND "lentiformis"[All Fields]) OR "nucleus lentiformis"[All Fields]) OR ("putamen"[MeSH Terms] OR "putamen"[All Fields]) OR ("globus pallidus"[MeSH Terms] OR ("globus"[All Fields] AND "pallidus"[All Fields]) OR "globus pallidus"[All Fields]) OR ("basal ganglia"[MeSH Terms] OR ("basal"[All Fields] AND "ganglia"[All Fields]) OR "basal ganglia"[All Fields])) AND ((transcranial[All Fields] AND ("ultrasonography"[MeSH Terms] OR "ultrasonography"[All Fields] OR "sonography"[All Fields])) OR (b-mode[All Fields] AND ("diagnostic imaging"[Subheading] OR ("diagnostic"[All Fields] AND "imaging"[All Fields]) OR "diagnostic imaging"[All Fields] OR "ultrasound"[All Fields] OR "ultrasonography"[MeSH Terms] OR "ultrasonography"[All Fields] OR "ultrasound"[All Fields] OR "ultrasonics"[MeSH Terms] OR "ultrasonics"[All Fields])) OR (transcranial[All Fields] AND ("diagnostic imaging"[Subheading] OR ("diagnostic"[All Fields] AND "imaging"[All Fields]) OR "diagnostic imaging"[All Fields] OR "ultrasound"[All Fields] OR "ultrasonography"[MeSH Terms] OR "ultrasonography"[All Fields] OR "ultrasound"[All Fields] OR "ultrasonics"[MeSH Terms] OR "ultrasonics"[All Fields]))) AND (parkinson[All Fields] OR ("multiple system atrophy"[MeSH Terms] OR ("multiple"[All Fields] AND "system"[All Fields] AND "atrophy"[All Fields]) OR "multiple system atrophy"[All Fields]) OR ("lewy body disease"[MeSH Terms] OR ("lewy"[All Fields] AND "body"[All Fields] AND "disease"[All Fields]) OR "lewy body disease"[All Fields] OR

("lewy"[All Fields] AND "body"[All Fields] AND "dementia"[All Fields]) OR "lewy body dementia"[All Fields]) OR ("supranuclear palsy, progressive"[MeSH Terms] OR ("supranuclear"[All Fields] AND "palsy"[All Fields] AND "progressive"[All Fields]) OR "progressive supranuclear palsy"[All Fields] OR ("progressive"[All Fields] AND "supranuclear"[All Fields] AND "palsy"[All Fields])) OR ("parkinsonian disorders"[MeSH Terms] OR ("parkinsonian"[All Fields] AND "disorders"[All Fields]) OR "parkinsonian disorders"[All Fields] OR ("parkinsonian"[All Fields] AND "syndrome"[All Fields]) OR "parkinsonian syndrome"[All Fields]) OR ("movement disorders"[MeSH Terms] OR ("movement"[All Fields] AND "disorders"[All Fields]) OR "movement disorders"[All Fields] OR ("movement"[All Fields] AND "disorder"[All Fields]) OR "movement disorder"[All Fields]))

Scopus (44 results):

ALL (nucleus AND lentiformis OR putamen OR globus AND pallidus OR basal AND ganglia AND transcranial AND sonography OR b-mode AND ultrasound OR transcranial AND parkinson OR multiple AND system AND atrophy OR lewy AND body AND dementia OR progressive AND supranuclear AND palsy OR movement AND disorder)

Duplicates: 4

Total abstracts to be assessed: 146
